# Alternative Global Health Security Indexes for Risk Analysis of COVID-19

**DOI:** 10.3390/ijerph17093161

**Published:** 2020-05-01

**Authors:** Chia-Lin Chang, Michael McAleer

**Affiliations:** 1 Department of Applied Economics and Department of Finance, National Chung Hsing University, Taichung 402, Taiwan; changchialin@email.nchu.edu.tw; 2 Department of Finance, Asia University, Taichung 41354, Taiwan; 3 Discipline of Business Analytics, University of Sydney Business School, Sydney, NSW 2006, Australia; 4 Econometric Institute, Erasmus School of Economics, Erasmus University Rotterdam, 3000 Rotterdam, The Netherlands; 5 Department of Economic Analysis and ICAE, Complutense University of Madrid, 28223 Madrid, Spain; 6 Institute of Advanced Sciences, Yokohama National University, Yokohama 240-8501, Japan

**Keywords:** global health security risk, pandemic, COVID-19, Pythagorean means, risk management, numerical rankings, ordinal rankings

## Abstract

Given the volume of research and discussion on the health, medical, economic, financial, political, and travel advisory aspects of the SARS-CoV-2 virus that causes the COVID-19 disease, it is essential to enquire if an outbreak of the epidemic might have been anticipated, given the well-documented history of SARS and MERS, among other infectious diseases. If various issues directly related to health security risks could have been predicted accurately, public health and medical contingency plans might have been prepared and activated in advance of an epidemic such as COVID-19. This paper evaluates an important source of health security, the Global Health Security Index (2019), which provided data before the discovery of COVID-19 in December 2019. Therefore, it is possible to evaluate how countries might have been prepared for a global epidemic, or pandemic, and acted accordingly in an effective and timely manner. The GHS index numerical scores are calculated as the arithmetic (AM), geometric (GM), and harmonic (HM) means of six categories, where AM uses equal weights for each category. The GHS Index scores are regressed on the numerical score rankings of the six categories to check if the use of equal weights of 0.167 in the calculation of the GHS Index using AM is justified, with GM and HM providing a check of the robustness of the arithmetic mean. The highest weights are determined to be around 0.244–0.246, while the lowest weights are around 0.186–0.187 for AM. The ordinal GHS Index is regressed on the ordinal rankings of the six categories to check for the optimal weights in the calculation of the ordinal Global Health Security (GHS) Index, where the highest weight is 0.368, while the lowest is 0.142, so the estimated results are wider apart than for the numerical score rankings. Overall, Rapid Response and Detection and Reporting have the largest impacts on the GHS Index score, whereas Risk Environment and Prevention have the smallest effects. The quantitative and qualitative results are different when GM and HM are used.

## 1. Introduction

There is no doubt that the COVID-19 disease, and the SARS-CoV-2 virus that causes it, have captured the world’s attention. With the exception of some countries where the leadership has tried to downplay, distort, and seemingly ignore its presence, most countries seem to have taken the coronavirus seriously from a public health and community safety perspective. Under such circumstances, it can be difficult to maintain a semblance of sanity when it is easy to entertain the alternative of panic.

At the time of writing, there is still no safe, reliable, efficient, and timely vaccine for the SARS-CoV coronavirus that caused SARS from 2002 to 2003, and for the MERS-CoV coronavirus that has continued to cause MERS since 2012. Therefore, it is difficult to feel optimistic about the discovery of a vaccine for COVID-19 in the foreseeable future.

For detailed medical studies on COVID-19, and government efforts to deal with the disease, see [1] Paules, Marston, and Fauci (2020), [2] del Rio and Malani (2020), [3] Parodi and Liu (2020), [4] Wang, Ng, and Brook (2020), [5] Wu and McCoogan (2020), [6] Sharfstein, Becker, and Mello (2020), [7] Wu, Chen, Cai et al. (2020), [8] Hoopman, Allegranzi, and Mehtar (2020), [9] Gostin, Hodge Jr., Wiley (2020), [10] Merchant and Lurie (2020), and [11] Yu, Ouyang, Chua et al. (2020), among others.

From a non-medical perspective, recent papers on risk management of COVID-19 include [12,13] McAleer (2020) and [14] Yang, Cheng, and Yue (2020).

Despite the volume of research and discussion on the health, medical, economic, financial, political, and travel advisory aspects of COVID-19, it is essential to enquire if an outbreak of the epidemic, which was belatedly classified as a global pandemic by the World Health Organization on 11 March 2020, might have been anticipated, given the well-documented history of SARS and MERS. 

For there to be a foreseeable and predictable outcome based on observable and credible data, rather than on possibly misguided perceptions and “hunches” that do not necessarily rely on provable facts, it is essential to consider a well-documented source of publicly available information about what might have been anticipated about epidemics such as COVID-19. If various issues directly related to health security risk could have been predicted accurately, public health and medical contingency plans might have been prepared and activated well in advance of the onset of a pandemic such as COVID-19.

The purpose of this paper is to critically evaluate an important source of health security, namely the Global Health Security Index (2019). The data in the 2019 Report were available before the discovery of COVID-19 as pneumonia of unknown form in December 2019. Therefore, it is possible to evaluate how countries might have been prepared for a global epidemic, or pandemic, and acted accordingly. 

The GHS Index numerical score rankings are obtained from [15] Global Health Security Index (2019), and are presented in Appendix A, while the GHS Index ordinal rankings are presented in Appendix B.

The remainder of the paper is as follows. Section 2 presents the Global Health Security (GHS) Index that is based on six broad categories. Section 3 provides an empirical evaluation of the numerical GHS scores and their respective rankings, as well as the corresponding ordinal rankings. Two regression models are estimated by least squares using both the numerical score and ordinal rankings, and optimal weights are assigned to each of the six categories in calculating the GHS Index. A conclusion and discussion of relevance are given in Section 4.

## 2. The Global Health Security (GHS) Index 

Among the 140 questions, the GHS Index “prioritizes not only countries’ capacities, but also the existence of functional, tested, proven capabilities for stopping outbreaks at the source” (https://www.ghsindex.org/about/#About-the-Index-Project-Team). 

The questions are organized across the following six categories:

1. Prevention: Prevention of the emergence or release of pathogens;
2. Detection and Reporting: Early detection and reporting for epidemics of potential international concern;
3. Rapid Response: Rapid response to and mitigation of the spread of an epidemic;
4. Health System: Sufficient and robust health system to treat the sick and protect health workers;
5. Compliance with International Norms: Commitments to improving national capacity, financing plans to address gaps, and adhering to global norms;
6. Risk Environment: Overall risk environment and country vulnerability to biological threats.

The GHS Index is a comprehensive assessment, developed as a collaboration between the Nuclear Threat Initiative, Johns Hopkins Center for Health Security, and the Economist Intelligence Unit, covering global health security capabilities in 195 countries. The GHS Index lists the countries that are best prepared for an epidemic or pandemic. “The average overall GHS Index score is 40.2 out of a possible 100. While high-income countries report an average score of 51.9, the Index shows that collectively, international preparedness for epidemics and pandemics remains very weak. Overall, the GHS Index finds severe weaknesses in a country’s abilities to prevent, detect, and respond to health emergencies; severe gaps in health systems; vulnerabilities to political, socioeconomic, and environmental risks that can confound outbreak preparedness and response; and a lack of adherence to international norms.” (https://www.ghsindex.org/report-model/). As part of China, Hong Kong was not included in the GHS Index as a country, while Taiwan was not included undoubtedly for political reasons. The data for the 195 countries are reported on pages 20–29 at: https://www.ghsindex.org/wp-content/uploads/2019/10/2019-Global-Health-Security-Index.pdf, which provides a numerical Average Overall score and separate numerical scores for each of the six categories. The seven numerical score rankings are obtained from Global Health Security Index (2019), and are reported in Appendix A, while the seven ordinal rankings are presented in Appendix B.

## 3. Empirical Evaluation 

This section provides an empirical evaluation of the numerical GHS scores according to seven data series, namely the numerical scores for Average Overall and 6 categories, and the respective numerical score rankings, as well as the corresponding ordinal rankings for the Average Overall and six categories. Two empirical models are estimated using the numerical score rankings and ordinal rankings, with the GHS Index regressed on the respective numerical score rankings and ordinal rankings of each of the six categories. 

The GHS Average Overall Index is the arithmetic mean numerical value that is calculated from the six numerical scores categories. The equal weight that is used for each category is 0.167. The abbreviations used are as follows: AO = Average Overall, PR = Prevention, DR = Detection and Reporting, RR = Rapid Response, HS = Health System, CO = Compliance, and RE = Risk Environment.

The descriptive statistics for the numerical score rankings are given in Table 1, which reports the mean, standard deviation, minimum and maximum values, and the range. The highest mean score is RE, and the lowest is HS. The highest standard deviation is DR, and the lowest is CO. The highest minimum is CO, and the lowest is HS. The highest maximum is DR, and the lowest HS. The largest range is DR and the lowest is CO.

It is instructive to present the 10 leading countries according to the AO numerical scores, together with the associated 6 category scores, namely:

(AO = 1) USA: PR = 1, DR = 1, RR = 1, HS = 1, CO = 1, RE = 19;
(AO = 2) UK: PR = 10, DR = 6, RR = 1, HS = 11, CO = 2, RE = 26;
(AO = 3) Netherlands: PR = 4, DR = 7, RR = 4, HS = 3, CO = 32, RE = 12;
(AO = 4) Australia: PR = 8, DR = 2, RR = 10, HS = 6, CO = 3, RE = 18;
(AO = 5) Canada: PR = 7, DR = 4, RR = 17, HS = 4, CO = 5, RE = 10;
(AO = 6) Thailand: PR = 3, DR = 15, RR = 5, HS = 2, CO = 12, RE = 93;
(AO = 7) Sweden: PR = 1, DR = 7, RR = 14, HS = 20, CO = 11, RE = 6;
(AO = 8) Denmark: PR = 5, DR = 7, RR = 19, HS = 5, CO = 28, RE = 17;
(AO = 9) South Korea: PR = 19, DR = 5, RR = 6, HS = 13, CO = 23, RE = 27;
(AO = 10) Finland: PR = 9, DR = 45, RR = 7, HS = 6, CO = 4, RE = 14.

The USA has the highest scores in five categories, but has an outlying score at 19 in Risk Environment (RE). The UK and Thailand also have apparent outliers in RE, with scores of 26 and 93, respectively. The Netherlands and Denmark have what seem to be outliers in Compliance (CO), at 32 and 28, respectively. Australia, Canada, and Sweden have relatively uniform scores in all six categories. South Korea has two outlying scores in CO and RE at 23 and 27, respectively. Finland has an outlier in Detection and Reporting (DR) at 45.

In the presence of outliers, the arithmetic mean can give a distorted measure of the central tendency of the individual components. Consequently, it is worth calculating the arithmetic mean (AM), geometric mean (GM), and harmonic mean (HM) using the numerical scores and ordinal rankings of each of the six categories for the 195 countries’ data for purposes of comparison. The GHS Index reported in the Global Health Security Index (2019) is calculated using the arithmetic mean, and is called AO. 

The Pythagorean means are special cases of the generalized, power, or Hölder means, which can extend the three means discussed above to weighted power means, such as the quadratic and cubic means. In the interest of keeping the empirical analysis manageable, only the three Pythagorean means will be used in the paper.

The three classical Pythagorean means satisfy the inequality.

(1) HM ≤ GM ≤ AM

The AM (=AO) of the numerical scores of the six categories is defined as:

(2) $$\mathrm{AO}=\frac{1}{6}\overset{6}{\underset{i=1}{\sum }}{\mathrm{GHS}}_{i}$$

where the subscript *i* = 1, 2, …, 6 represents PR, DR, RR, HS, CO and RE, respectively. The AM score might be referred to as GHS(AM), but we will continue to use AO, as given in the Global Health Security Index (2019). 

Two new alternative GHS mean scores are as follows. The geometric mean of the GHS scores, which is an arithmetic mean of the logarithms of the six GHS scores when all the observations are positive, is defined as:

(3) $$\mathrm{GM}={\left(\overset{6}{\underset{i=1}{\prod }}{\mathrm{GHS}}_{i}\right)}^{1/6}$$

where the subscript *i* = 1, 2, …, 6 represents PR, DR, RR, HS, CO and RE, respectively.

The harmonic mean, which measures the reciprocal of the arithmetic mean of the reciprocals of the six GHS scores, is defined as:

(4) $$\mathrm{HM}=6/\left(\overset{6}{\underset{i=1}{\sum }}{\frac{1}{\mathrm{GHS}}}_{i}\right)$$

where the subscript *i* = 1, 2, …, 6 represents PR, DR, RR, HS, CO and RE, respectively. 

In the empirical analysis, the new GHS average scores, GM and HM will be analyzed together with AO. According to the inequality in Equation (1), the three means satisfy.

(5) HM ≤ GM ≤ AO

If the rankings of all three means in Equation (5) are similar, according to the pairwise correlation coefficients, the use of AO would seem to be reasonable, although arbitrary. However, if the pairwise correlations are dissimilar, then the use of AO would be questionable, especially given the outliers among the six GHS rankings. This is especially the case when the chosen rankings would depend on an arbitrary selection of a Pythagorean mean.

Returning to Table 1, the means satisfy the condition in Equation (5), as do the minimum values of the numerical scores. The standard deviations are in reverse order to the respective means, as is the range. The maximum values of the numerical scores are similar.

The correlations of the numerical score rankings are given in Table 2. The correlations among AO, GM, and HM are high in the range (0.982, 0.997), with GM and HM having the highest correlation at 0.997. The correlation between DR and RR is very high at 0.987. The next highest correlations are between AO and PR, HS, DR and RR, with all values above 0.89. The correlations of GM and HM with these categories are similar to those of AO. The lowest correlations are between RE and CO, DR and RR, with all values below 0.44.

The correlations of the ordinal rankings are given in Table 3, which qualitatively match the results in Table 2. The correlations among AO, GM, and HM are high in the range (0.950, 0.987), with GM and HM having the highest correlation at 0.987. The correlation between DR and RR is 0.999, which means that the two categories are virtually identical. The next highest correlations are between AO and HS, DR, RR and PR, with all values above 0.88. The correlations of GM and HM with these categories mirror those of AO. The lowest correlations are between RE and CO, RR and DR, with all values below 0.39.

The numerical score GHS Index is regressed on the numerical score rankings of the six categories in Table 4 to check if equal weights in the calculation of the GHS Index are justified. Given the high correlation between DR and RR in Table 2, it is not surprising that RR is statistically insignificant in the first column in Table 4. Each DR and RR are deleted in the second and third columns in Table 4, where the other variable is found to be statistically significant. The highest weights in each case are determined to be RR at 0.325, while the lowest weights are for PR at 0.186 and RE at 0.128. Therefore, Rapid Response has a large impact on the GHS Index numerical score.

The quantitative and qualitative results for GM and HM in Table 5 and Table 6 are quite different from those of AO in Table 4. Both DR and RR are significant for GM, whereas RR is insignificant for HM. The highest weight for GM is RR at 0.42, while the lowest weights are RE at 0.109 and CO at 0.13, which are markedly different from the weights for AO. The highest weights for HM is RR at 0.398 and HS at 0.366, while the lowest weights are RE at 0.076 and CO at 0.096, which are substantially lower than the corresponding weights for AO, as well as lower than for GM.

Overall, the range in the weights is much greater for both GM and HM than they are for AO, although RR has the highest weights for each of the three means.

The ordinal GHS Index is regressed on the ordinal rankings of the six categories in Table 7 to check for the optimal weights in the calculation of the ordinal GHS Index. Given the correlation of 0.999 between DR and RR in Table 3, it is not surprising that both categories are insignificant for AO in the first column when they appear simultaneously, while RR is only marginally significant. Deleting DR and RR in turn leads to the estimates in the second and third columns in Table 7, respectively, which show that the estimates for AO are identical, a result that is mirrored for GM and HM. With AO as the dependent variable, the highest weights are for DR and RR at 0.368, while the lowest is for RE at 0.142. 

Broadly similar results hold for GM and HM in Table 8 and Table 9, respectively. The categories DR and RR also have the highest weights for GM and HM, but with higher numerical values of 0.382–0.383 for GM, and a lower numerical value of 0.341 for HM. However, unlike the case for AO where the lowest weight was for RE at 0.142, the lowest weight for GM is PR at 0.175. The lowest weight for HM is also PR, but at much lower weights of 0.118–0.119. It is clear that the ordinal rankings differ more widely across AO, GM and HM than they did for the GHS numerical score rankings.

Overall, Rapid Response and Detection and Reporting have strong impacts on the GHS Index ordinal ranking, regardless of whether the mean is AO, GM, or HM. While Risk Environment has the smallest impact on the GHS Index ordinal score for AO, Prevention has the smallest impact for GM and HM.

## 4. Conclusions 

Given the volume of research and discussion on the health, medical, economic, financial, political, and travel advisory aspects of COVID-19, it is essential to enquire if an outbreak of the epidemic might have been anticipated, in light of the well-documented history of SARS and MERS. If various issues directly related to health security risks could have been predicted accurately, public health and medical contingency plans might have been prepared and activated well in advance of the onset of an epidemic such as COVID-19.

In this light, this paper critically evaluated an important source of health security, namely the Global Health Security Index (2019), which provided data before the discovery of COVID-19 in January 2020. Therefore, it is possible to evaluate how countries might have been prepared for a global epidemic, or pandemic, and acted accordingly.

The GHS Index numerical score is the arithmetic mean of the data for six categories, and hence uses equal weights for each category. The AO of the GHS Index score was regressed on the numerical score rankings of the six categories to check if the use of equal weights of 0.167 in the calculation of the GHS Index was justified. The highest weights were determined to be around 0.244–0.246, while the lowest weights were around 0.186–0.187.

Two alternative mean scores, namely the geometric mean (GM) and harmonic mean (HM), were also calculated from the numerical GHS Index scores. In addition to presenting alternative means of the GHS scores, they also provide a check of the robustness of the arithmetic mean score (AO) in the Global Health Security Index (2019). Although the three means suggested that Rapid Response had the largest impact, albeit with different weights, AO found the smallest impact from Prevention and Risk Environment, whereas both GM and HM found Compliance and Risk Environment had the smallest impacts.

The ordinal GHS Index was regressed on the ordinal rankings of the six categories to check for the optimal weights in the calculation of the ordinal GHS Index. The highest weight was 0.368, while the lowest was 0.142, so the estimated results are wider apart at 0.226 than for the numerical score rankings. The range was smaller for GM at 0.180 and for HM at 0.199.

Overall, Rapid Response and Detection and Reporting have the largest impacts on the GHS Index score, regardless of whether AO, GM, or HM were used, albeit with different weights. Risk Environment has the smallest impact on the GHS Index score when AO is used, whereas Prevention has the lowest impacts for GM and HM.

In preparing for an epidemic or pandemic, the order and importance of risk factors need to be known so that public health and medical contingency plans can be coordinated and activated effectively and in a timely manner. In such an environment, it is revealing that Rapid Response and Detection and Reporting have the largest impacts.

## Figures and Tables

**Table 1:** Descriptive statistics for numerical score rankings.

Score	Mean	Std. Dev.	Min	Max	Range
PR	34.73	16.96	1.9	83.1	81.2
DR	41.88	23.81	2.7	98.2	95.5
RR	38.43	15.12	11.3	91.9	80.6
HS	26.43	16.87	0.3	73.8	73.5
CO	48.48	12.64	23.3	85.3	62.0
RE	55.03	16.20	15.9	87.9	72.0
AO	40.20	14.52	16.2	83.5	67.3
GM	38.21	15.58	10.2	84.7	74.5
HM	35.69	16.71	1.7	84.3	82.6

Notes: 195 observations; PR = Prevention, DR = Detection and Reporting, RR = Rapid Response, HS = Health System, CO = Compliance, RE = Risk Environment, AO = Average Overall. GM is the geometric mean GHS score, and HM is the harmonic mean GHS score; the mean AO score is taken from GHS Index (2019).

**Table 2:** Correlations of numerical score rankings.

Score	PR	DR	RR	HS	CO	RE	AO	GM	HM
PR	1								
DR	0.772 *	1							
RR	0.774 *	0.987 *	1						
HS	0.843 *	0.741 *	0.747 *	1					
CO	0.636 *	0.633 *	0.633 *	0.583 *	1				
RE	0.576 *	0.426 *	0.430 *	0.624 *	0.311 *	1			
AO	0.916 *	0.894 *	0.893 *	0.914 *	0.736 *	0.647 *	1		
GM	0.920 *	0.916 *	0.915 *	0.914 *	0.714 *	0.631 *	0.989 *	1	
HM	0.918 *	0.900 *	0.899 *	0.928 *	0.693 *	0.620 *	0.982 *	0.997 *	1

Notes: * denotes significance at 1%; 195 observations; PR = Prevention, DR = Detection and Reporting, RR = Rapid Response, HS = Health System, CO = Compliance, RE = Risk Environment, AO = Average, Overall. GM is the geometric mean GHS score, and HM is the harmonic mean GHS score.

**Table 3:** Correlations of ordinal rankings.

Rank	PR	DR	RR	HS	CO	RE	AO	GM	HM
**PR**	1								
**DR**	0.750 *	1							
**RR**	0.750 *	0.999 *	1						
**HS**	0.813 *	0.720 *	0.719 *	1					
**CO**	0.594 *	0.598 *	0.598 *	0.520 *	1				
**RE**	0.550 *	0.389 *	0.388 *	0.580 *	0.285 *	1			
**AO**	0.894 *	0.885 *	0.885 *	0.887 *	0.703 *	0.612 *	1		
**GM**	0.886 *	0.884 *	0.884 *	0.869 *	0.726 *	0.658 *	0.979 *	1	
**HM**	0.845 *	0.840 *	0.840 *	0.838 *	0.711 *	0.667 *	0.950 *	0.987 *	1

Notes: * denotes significance at 1%; 195 observations; PR = Prevention, DR = Detection and Reporting, RR = Rapid Response, HS = Health System, CO = Compliance, RE = Risk Environment, AO = Average, Overall. GM is the geometric mean GHS score, and HM is the harmonic mean GHS score.

**Table 4:** Numerical scores of AO regressed on six category numerical score rankings.

Variables	AO	AO	AO
PR	0.186 ** (0.016)	0.192 ** (0.017)	0.186 ** (0.016)
DR	0.202 ** (0.029)		0.212 ** (0.009)
RR	0.017 (0.044)	0.325 ** (0.017)	
HS	0.245 ** (0.015)	0.244 ** (0.016)	0.246 ** (0.015)
CO	0.191 ** (0.013)	0.194 ** (0.014)	0.191 ** (0.013)
RE	0.129 ** (0.010)	0.128 ** (0.010)	0.187 ** (0.010)
Intercept	1.813 * (0.867)	−1.864 ** (0.743)	2.013 ** (0.649)
R-squared	0.987	0.984	0.987
F statistic	2441.94 **	1653.93 **	2908.46 **

Notes: White’s heteroskedasticity-robust standard errors are given in parentheses; * and ** denote significance at 5% and 1%, respectively; 196 observations; PR = Prevention, DR = Detection and Reporting, RR = Rapid Response, HS = Health System, CO = Compliance, RE = Risk Environment, AO = Average Overall.

**Table 5:** Numerical score of GM regressed on six category numerical score rankings.

Variables	GM	GM	GM
PR	0.213 * (0.009)	0.220 * (0.011)	0.213 * (0.009)
DR	0.228 * (0.015)		0.272 * (0.006)
RR	0.072 * (0.023)	0.420 * (0.013)	
HS	0.255 * (0.009)	0.253 * (0.010)	0.257 * (0.010)
CO	0.130 * (0.007)	0.134 * (0.009)	0.130 * (0.008)
RE	0.110 ** (0.007)	0.109 * (0.008)	0.110 * (0.007)
Intercept	−0.590 * (0.589)	−4.749 * (0.639)	0.253 * (0.515)
R-squared	0.996	0.993	0.996
F statistic	7255.06*	2437.79*	7496.75 *

Notes: White’s robust standard errors are given in parentheses; * denotes significance at 1%; 196 observations; PR = Prevention, DR = Detection and Reporting, RR = Rapid Response, HS = Health System, CO = Compliance, RE = Risk Environment, AO = Average Overall.

**Table 6:** Numerical score of HM regressed on six category numerical score rankings.

Variables	HM	HM	HM
PR	0.229 * (0.017)	0.236 * (0.017)	0.229 * (0.017)
DR	0.244 * (0.028)		0.259 * (0.013)
RR	0.025 (0.042)	0.398 * (0.022)	
HS	0.365 * (0.022)	0.363 * (0.022)	0.366 * (0.022)
CO	0.096 * (0.015)	0.100 * (0.016)	0.096 * (0.015)
RE	0.078 * (0.015)	0.076 * (0.015)	0.078 * (0.015)
Intercept	−2.022 (1.286)	−6.468 * (1.187)	−1.731 (1.088)
R-squared	0.986	0.983	0.986
F statistic	2361.52 *	1418.52 *	2830.30 *
			

Notes: White’s robust standard errors are given in parentheses; * denote significance at 1%; 196 observations; PR = Prevention, DR = Detection and Reporting, RR = Rapid Response, HS = Health System, CO = Compliance, RE = Risk Environment, AO = Average Overall.

**Table 7:** Ordinal score of AO regressed on six category ordinal rankings.

Variable	AO	AO	AO
PR	0.214 * (0.028)	0.213 * (0.028)	0.214 * (0.028)
DR	−1.027 (0.804)		0.368 * (0.024)
RR	1.392 (0.800)	0.368 * (0.024)	
HS	0.278 * (0.027)	0.277 * (0.028)	0.277 * (0.028)
CO	0.172 * (0.017)	0.172 * (0.017)	0.172 * (0.017)
RE	0.142 * (0.017)	0.142 * (0.017)	0.142 * (0.017)
Intercept	−16.82 * (1.538)	−16.834 * (1.555)	−16.828 * (1.565)
R-squared	0.970	0.970	0.970
F statistic	1610.21 *	1787.49 *	1758.08 *

Notes: White’s heteroskedasticity-robust standard errors are given in parentheses; * denotes significance at 1%; 196 observations; PR = Prevention, DR = Detection and Reporting, RR = Rapid Response, HS = Health System, CO = Compliance, RE = Risk Environment, AO = Average Overall.

**Table 8:** Ordinal score of GM regressed on six category ordinal rankings.

Variable	GM	GM	GM
PR	0.146 * (0.023)	0.146 * (0.023)	0.146 * (0.023)
DR	−0.434 (0.494)		0.326 * (0.016)
RR	0.758 (0.489)	0.326 * (0.016)	
HS	0.168 * (0.020)	0.168 * (0.020)	0.168 * (0.020)
CO	0.186 * (0.013)	0.186 * (0.013)	0.186 * (0.013)
RE	0.196 * (0.015)	0.196 * (0.015)	0.196 * (0.015)
Intercept	−8.701 * (1.029)	−8.707 * (1.027)	−8.705 * (1.028)
R-squared	0.984	0.984	0.984
F statistic	3060.98 *	3342.36 *	3298.33 *

Notes: White’s robust standard errors are given in parentheses; * denotes significance at 1%; 196 observations; PR = Prevention, DR = Detection and Reporting, RR = Rapid Response, HS = Health System, CO = Compliance, RE = Risk Environment, AO = Average Overall.

**Table 9:** Ordinal scores of HM regressed on six category ordinal rankings.

Variable	HM	HM	HM
PR	0.105 * (0.053)	0.105 * (0.053)	0.105 * (0.053)
DR	−1.196 (1.153)		0.304 ** (0.032)
RR	1.496 (1.143)	0.304 ** (0.032)	
HS	0.172 ** (0.047)	0.171 ** (0.047)	0.171 ** (0.047)
CO	0.213 ** (0.029)	0.213 ** (0.030)	0.213 ** (0.030)
RE	0.242 ** (0.035)	0.241 ** (0.035)	0.241 ** (0.035)
Intercept	−16.687 ** (2.051)	−16.701 ** (2.045)	−16.694 ** (2.046)
R-squared	0.970	0.924	0.924
F statistic	1610.21 **	765.06 **	758.56 **

Notes: White’s robust standard errors are given in parentheses; * and ** denotes significance at 5% and 1%, respectively; 196 observations; PR = Prevention, DR = Detection and Reporting, RR = Rapid. Response, HS = Health System, CO = Compliance, RE = Risk Environment, AO = Average Overall.

## Appendix A

**Table A1:** GHS Index Numerical Score Rankings for 195 Countries.

Score	AO	GM	HM	PR	DR	RR	HS	CO	RE
United States	83.5	84.70	84.32	83.1	98.2	91.9	73.8	85.3	78.2
United Kingdom	77.9	73.26	72.72	68.3	87.3	71.5	59.8	81.2	74.7
Netherlands	75.6	72.92	72.45	73.7	86	67.7	70.2	61.1	81.7
Australia	75.5	76.94	76.27	68.9	97.3	79.7	63.5	77	79.4
Canada	75.3	77.89	77.37	70	96.4	79.1	67.7	74.7	82.7
Thailand	73.2	68.90	68.39	75.7	81	61.9	70.5	70.9	56.4
Sweden	72.1	71.97	70.58	81.1	86	67.1	49.3	71.3	84.5
Denmark	70.4	71.98	71.52	72.9	86	69.2	63.8	62.6	80.3
South Korea	70.2	69.84	68.87	57.3	92.1	78.6	58.7	64.3	74.1
Finland	68.7	65.36	64.52	68.5	61.6	49.7	60.8	75.4	81.1
France	68.2	67.22	66.61	71.2	75.3	58.1	60.9	58.6	83
Slovenia	67.2	65.56	65.01	67	73.7	55.1	54.9	72.1	73.7
Switzerland	67	61.26	60.26	52.7	59.1	48	62.5	65.6	86.2
Germany	66	67.07	65.87	66.5	84.6	65.9	48.2	61.9	82.3
Spain	65.9	65.59	64.83	52.9	83	64.6	59.6	61.1	77.1
Norway	64.6	63.05	62.04	68.2	58.6	47.9	58.5	64.4	87.1
Latvia	62.9	64.23	62.28	56	97.3	79.3	47.3	51.1	67.2
Malaysia	62.2	60.59	60.06	51.4	73.2	54.7	57.1	58.5	72
Belgium	61	61.90	61.39	63.5	62.5	50.2	60.5	59.7	78.2
Portugal	60.3	56.47	55.68	52.8	50.5	45.4	55	63	77.3
Japan	59.8	58.97	57.99	49.3	70.1	52	46.6	70	71.7
Brazil	59.7	56.55	55.19	59.2	82.4	63.3	45	41.9	56.2
Ireland	59	60.55	58.93	63.9	78	60.2	40.2	52.8	77.4
Singapore	58.7	55.45	54.19	56.2	64.5	50.6	41.4	47.3	80.9
Argentina	58.6	58.61	57.55	41.4	74.9	57.7	54.9	68.8	60
Austria	58.5	60.27	59.08	57.4	73.2	54.8	46.6	52.8	84.6
Chile	58.3	56.19	55.00	56.2	72.7	54.3	39.3	51.5	70.1
Mexico	57.6	56.75	55.77	45.5	71.2	52.2	46.9	73.9	57
Estonia	57	56.86	54.01	47.6	77.6	58.4	31.6	67.6	73.3
Indonesia	56.6	54.81	53.69	50.2	68.1	51.7	39.4	72.5	53.7
Italy	56.2	56.95	55.20	47.5	78.5	61.3	36.8	61.9	65.5
Poland	55.4	55.94	55.53	50.9	61.7	49.9	48.9	58.9	67.9
Lithuania	55	57.85	55.12	43.5	81.5	62.9	34.4	72.1	67.8
South Africa	54.8	52.79	50.59	44.8	81.5	62.8	33	46.3	61.8
Hungary	54	52.84	51.79	56.4	55.5	47.3	36.6	58.9	68.2
New Zealand	54	49.20	47.30	55	36.7	33.9	45.2	59.4	77.2
Greece	53.8	55.01	53.66	54.2	78.4	60.7	37.6	49.1	58.2
Croatia	53.3	56.72	56.00	55.2	72.3	53.6	46.5	49.1	68.2
Albania	52.9	51.87	50.56	43.8	74.3	56.5	35.9	53	55.7
Turkey	52.4	51.42	50.87	56.9	45.6	42.9	45.7	64.3	56.5
Serbia	52.3	50.43	50.15	48.8	46.2	43.8	56.6	49.7	59.2
Czech Republic	52	51.92	50.82	51.1	50.7	46.4	37.4	58.9	74
Georgia	52	54.23	53.17	53.2	75	57.8	38.3	56	51.4
Armenia	50.2	47.13	45.04	56.7	60.8	49	25.7	50.1	50.4
Ecuador	50.1	50.99	49.75	53.9	71.2	52.4	35.2	43.5	57.1
Mongolia	49.5	50.54	48.14	37.6	77.3	58.2	30.8	52.6	60.8
Kyrgyz Republic	49.3	46.85	44.24	29.7	64.7	50.8	29.8	64.8	56.1
Saudi Arabia	49.3	51.95	50.42	34.3	74.4	56.9	44.8	50.6	59.7
Peru	49.2	45.91	44.91	43.2	38.3	34.6	45	63	57.7
Vietnam	49.1	48.60	46.80	49.5	57.4	47.5	28.3	64.6	53.4
China	48.2	47.58	47.10	45	48.5	44.8	45.7	40.3	64.4
Slovakia	47.9	49.78	48.82	53.5	46	43.2	37.9	52.8	71.5
Philippines	47.6	47.72	46.99	38.5	63.6	50.4	38.2	49.8	50.3
Israel	47.3	48.46	47.77	44	52.4	46.6	42.2	41.5	68.8
Kenya	47.1	45.79	41.83	45.9	68.6	51.8	20.7	67.1	40.7
United Arab Emirates	46.7	41.25	37.88	49.3	31.6	30.1	22.9	63.4	72.4
India	46.5	44.78	44.36	34.9	47.4	44	42.7	47.7	54.4
Iceland	46.3	44.05	42.41	35.3	37.2	34.2	46.4	43.2	81.2
Kuwait	46.1	44.81	44.25	40.9	47.5	44	36.5	42.2	61.5
Romania	45.8	46.69	45.84	48.9	42.8	39.2	36.7	52.4	65.7
Bulgaria	45.6	49.99	48.96	37.6	53.3	46.6	41	61.5	66.3
Costa Rica	45.1	45.58	43.14	44.2	56	47.3	24.8	43.1	71.7
Russia	44.3	41.03	40.31	42.9	34.1	32.1	37.6	52.6	51.4
Uganda	44.3	37.10	30.75	42.7	50.3	45.1	11.6	65.4	35.5
Colombia	44.2	42.69	41.89	37.2	41.7	37.1	34.3	60.1	51
El Salvador	44.2	42.06	38.19	22.1	73.9	55.5	25.2	50.5	48
Luxembourg	43.8	44.84	42.76	31	41.7	37.1	37.9	52.8	84.7
Montenegro	43.7	45.45	44.01	36.5	55.4	47	29.5	53.5	58.8
Morocco	43.7	41.37	40.00	34.6	56.8	47.3	29.5	32.7	55.9
Panama	43.7	42.61	41.82	40.5	44.6	41.9	35.1	35.3	63.8
Liechtenstein	43.5	39.80	36.02	43.1	22.9	25.9	31.1	56.9	87.9
Myanmar	43.4	39.57	36.40	30.3	59.2	48.6	19.5	59.1	38.2
Laos	43.1	37.73	33.14	18.9	70.4	52	19.4	45.9	46.8
Lebanon	43.1	40.67	38.22	27.3	62	50.1	23.8	49.3	45.5
Nicaragua	43.1	42.22	41.91	41.7	39.9	34.9	45.9	51.8	41
Oman	43.1	41.22	39.28	35.3	41.1	36.2	25.4	56	65.7
Cyprus	43	43.31	40.58	46.4	44.9	42.3	21.9	49.1	69.6
Moldova	42.9	44.47	44.05	46.5	42.9	39.9	36.4	56.7	47.1
Bosnia and Herzegovina	42.8	40.16	39.92	36.7	41.7	37.3	38.3	37.8	50.8
Jordan	42.1	40.06	38.94	31.8	42.9	40.2	27.8	48.6	55.8
Uruguay	41.3	38.52	36.40	44	33.5	31.3	24.1	39.3	74.8
Qatar	41.2	37.63	36.42	33.1	32.7	30.4	38.8	32.7	68
Kazakhstan	40.7	40.12	37.76	58.8	28.2	28.6	28	52.8	59.5
Ethiopia	40.6	36.85	35.71	36.8	33.7	31.5	29	65.8	33.6
Bhutan	40.3	39.47	38.60	35.5	42.8	39.5	27.9	39.7	56.9
Madagascar	40.1	34.35	32.51	30.1	41.9	37.8	19.2	55.4	32.4
Egypt	39.9	36.39	33.07	36.5	41.5	36.6	15.7	46.4	57.5
Bahrain	39.4	38.33	37.01	36	45.8	43.2	27.7	27.8	57.8
Cambodia	39.2	36.03	30.12	28.6	57.7	47.8	12	60	38.5
North Macedonia	39.1	39.38	38.17	37	41.7	36.8	25.4	44.8	57.7
Dominican Republic	38.3	34.21	31.41	30.5	37.1	34.1	16.1	43.5	59.3
Sierra Leone	38.2	35.95	34.47	25	45.8	43	25.3	52.8	32.8
Zimbabwe	38.2	37.56	33.00	31.4	65.6	51.5	14.7	45.9	39.2
Ukraine	38	36.96	35.65	38.1	36.5	33.4	23	55.1	43.3
Senegal	37.9	33.75	31.43	25.4	35.1	32.6	18.5	57	48.2
Nigeria	37.8	35.11	33.00	26.3	44.6	42	19.9	56.7	33.7
Iran	37.7	37.77	37.15	44.7	37.7	34.5	34.6	28.7	50.3
Malta	37.3	37.85	35.65	35	32.9	30.5	23.6	49.1	72.3
Trinidad and Tobago	36.6	30.17	26.60	28.1	14.7	21.7	23.7	55.1	64.4
Suriname	36.5	32.27	29.77	23.3	36.7	33.9	16.5	44.8	52.7
Tanzania	36.4	32.06	24.76	33.5	42	38	8.2	55.4	44.7
Bolivia	35.8	34.39	31.14	44	33.1	30.9	14.9	48.5	50.9
Paraguay	35.7	36.75	35.98	39.5	34.6	32.4	28.2	35.3	55.9
Namibia	35.6	34.07	27.79	32	46	43.5	10.1	44.2	54.7
Côte d’Ivoire	35.5	35.43	32.66	27.3	44.5	41.6	17.1	53.6	42.7
Ghana	35.5	35.75	34.74	32.2	40.5	35.3	23.4	38	51
Pakistan	35.5	33.49	31.76	24.1	41.7	36.7	19.9	49.7	38.7
Belarus	35.3	31.12	29.61	19.4	28.9	29.2	40.6	25.8	53
St. Lucia	35.3	27.54	19.74	22.8	30.3	29.5	6.3	54.7	62.1
Cuba	35.2	31.46	25.88	41.4	10.5	20.7	37.4	49.8	57.8
Liberia	35.1	29.41	26.14	14.3	29.1	29.2	19.9	71.5	37.4
Nepal	35.1	31.87	30.81	43.7	22	25.9	28.1	33.5	44.7
Bangladesh	35	36.06	31.99	27.3	50.9	46.6	14.7	52.5	44
Mauritius	34.9	33.09	29.77	27.3	42.3	39.1	15.1	29.1	66.2
Cameroon	34.4	33.50	32.03	28.2	35.6	32.7	21.4	59.9	33.6
Uzbekistan	34.3	31.32	27.85	42.6	19.4	24.7	16	60.5	47.8
Azerbaijan	34.2	35.68	33.29	30.8	45	42.4	17.9	36.2	54.2
Gambia	34.2	33.29	31.88	22	36.9	34.1	23.5	44.2	47.3
Rwanda	34.2	34.23	33.65	33.8	36	33.1	24.1	38	43.6
Sri Lanka	33.9	34.49	31.57	24.2	43	40.5	16.9	41.7	56.7
Maldives	33.8	30.09	27.85	21.8	25.5	27.8	18.1	45.5	58.3
Tunisia	33.7	31.52	30.51	31.7	26.3	28.4	24	31	55.7
St. Vincent and The Grenadine	33	29.80	26.71	20	20.6	25	19	58	61.7
Micronesia	32.8	24.83	22.75	21	14.2	21.7	18.8	36.3	53.1
Guatemala	32.7	32.26	27.13	21.2	50	45	11.4	42.2	49.1
Guinea	32.7	30.94	23.67	27	57.2	47.5	8	47.8	31.3
Monaco	32.7	29.13	24.58	11.1	23.3	26	31	35.3	83.1
Brunei	32.6	30.75	29.12	24.8	30.5	29.7	24.2	23.3	66.7
Togo	32.5	30.75	25.58	23.7	46.8	43.8	10	46.3	37.6
Afghanistan	32.3	32.69	30.47	23.5	44.8	42.1	21	56.3	23.3
Tajikistan	32.3	28.79	27.89	26.7	24.1	26.5	20.5	42.6	38.2
Niger	32.2	34.52	33.29	32.5	44.4	41.3	21.9	45.5	28.5
Barbados	31.9	27.39	21.64	33.3	19.1	24.3	8.5	46	69.9
seychelles	31.9	29.64	24.03	9.8	33.4	31.1	19.9	47.1	71.1
Belize	31.8	29.67	24.76	30	30.4	29.5	9.7	49.3	53
Turkmenistan	31.8	31.95	29.42	31	38.6	34.8	14.4	39.3	45.1
Guyana	31.7	27.47	24.31	27.9	20.3	24.8	12.3	49.3	50.5
Haiti	31.5	31.67	26.74	31.5	48.3	44.7	10.6	48.4	28.9
Botswana	31.1	29.72	26.28	22	28.2	28.9	13.3	46.3	62.4
San Marino	31.1	30.38	27.29	22.3	33.9	31.9	16.2	25	80.5
Eswatini (Swaziland)	31.1	26.75	20.02	35.7	25.5	27.2	6.5	46.6	48.9
Bahamas	30.6	25.96	20.68	24.7	21.8	25.5	7.9	46	61.4
Andorra	30.5	24.46	19.74	27.9	14.2	21.7	9.2	32.4	83.5
Lesotho	30.2	27.60	25.95	24.4	18	23.9	20.6	45.9	44.5
Burkina Faso	30.1	24.14	17.53	18	33.3	30.9	5.6	44.8	42.6
Cabo Verde	29.3	24.11	20.11	27.9	9.3	20.6	16.1	33.9	67.4
Antigua and Barbuda	29	24.60	18.87	17.8	19.1	24.5	7.4	55.1	65.2
Jamaica	29	26.49	22.41	20.1	24.3	26.8	10	43.1	61.2
Mali	29	26.69	24.44	23.4	25.5	27.3	13	53.2	32.1
Benin	28.8	22.69	16.66	16.5	24.2	26.6	5.6	53.6	42.8
Chad	28.8	24.31	19.02	23.2	36.5	33.7	6.6	46.2	23.7
Zambia	28.7	27.85	26.80	24.5	21.9	25.5	20.3	38	44.2
Mozambique	28.1	29.44	28.08	26.5	29.3	29.3	17	43.8	38.4
Malawi	28	27.72	25.86	25.5	23.3	26.2	15.3	50.7	37.6
Papua New Guinea	27.8	23.73	19.95	10	31.8	30.2	11.6	41.4	38.7
Honduras	27.6	26.38	23.98	21.6	27.7	28.4	12	41.8	39.5
Grenada	27.5	22.10	17.35	8.6	18.6	24.2	10.3	46.4	62.9
Mauritania	27.5	26.31	22.49	9.9	39.5	34.8	17	36.3	39.5
Central African Republic	27.3	21.47	20.09	18	17.7	23.6	12.8	44.2	23
Comoros	27.2	24.28	20.92	19.2	23.2	26	9.4	51.6	36.5
Congo (Democratic Republic)	26.5	23.71	21.84	24	25.1	27.1	11.8	45.9	20.1
Samoa	26.4	21.96	18.51	20.2	14.1	21.1	9.2	30.7	66.1
St. Kitts and Nevis	26.2	20.00	14.89	8.7	15	23	7.1	46.4	64.8
Sudan	26.2	20.48	16.92	31.8	7	18.7	14.3	37.6	33
Vanuatu	26.1	22.06	17.21	24.5	15	21.8	6.6	38	57.4
Timor-Leste	26	23.78	20.99	18.2	25.7	28.3	9.7	33.9	41.5
Iraq	25.8	26.76	24.23	22.1	42.2	38.7	11.8	29.5	29.2
Fiji	25.7	21.99	18.09	24.6	16.4	23.1	7.5	27.4	59.1
Libya	25.7	25.95	22.32	23.2	36	33.2	9.1	31	39
Angola	25.2	24.04	21.47	24	17.9	23.6	10.9	41.4	42.2
Tonga	25.1	21.54	17.56	19.8	15	22.4	7.5	33.9	59
Dominica	24	19.57	15.48	11.2	10.7	20.7	8.5	49.3	54
Algeria	23.6	22.40	19.98	25.7	12	20.9	13.1	29.1	51.4
Congo (Brazzaville)	23.6	17.79	13.18	17.6	7	18.9	6.3	56.8	38.1
Djibouti	23.2	21.27	18.65	16.3	17	23.2	9.3	36.3	42.7
Venezuela	23	20.79	17.78	23.5	8.7	19	12.9	42.2	38.2
Burundi	22.8	19.58	17.15	25.1	11.4	20.8	8.9	37.6	28.3
Eritrea	22.4	22.25	19.88	23.4	17.2	23.4	9.7	40	33.2
Palau	21.9	17.55	14.20	8.2	8.8	19.6	11.5	32	56.2
South Sudan	21.7	20.80	20.00	22.6	15.9	23	13.6	32.6	22.1
Tuvalu	21.6	18.84	15.88	13.1	8.7	19.5	12	28.6	58.7
Nauru	20.8	15.45	11.35	9.1	4.4	17.5	12	32	50.6
Solomon Islands	20.7	17.76	14.52	8.4	8.7	19.6	12.4	40.1	44
Niue	20.5	15.39	11.19	11	4.4	17.4	9.1	29.9	57.9
Cook Islands	20.4	18.55	15.82	10.9	8.8	19.7	14.3	29.9	50.5
Gabon	20	16.61	13.29	10.8	6.1	18.2	11.2	36.5	42.8
Guinea-Bissau	20	18.18	13.71	14	23.4	26.4	4.6	37.6	24.1
Syria	19.9	14.82	9.58	18.4	2.7	11.3	24.4	26.1	29.6
Kiribati	19.2	14.39	10.58	10.7	4.4	17.8	7.3	32.3	45
Yemen	18.5	16.43	14.08	15.1	9	20.1	7.6	40.3	23.5
Marshall Islands	18.2	10.93	5.99	1.9	4.4	17.6	7.2	30.7	52.3
São Tomé and Príncipe	17.7	12.50	8.04	8.2	2.7	16	7.2	33.5	44.6
North Korea	17.5	17.58	15.16	19	7	18.7	12.2	27.3	35.6
Somalia	16.6	10.25	1.68	15.8	21.5	25.1	0.3	28.5	15.9
Equatorial Guinea	16.2	10.17	5.65	1.9	4.4	18.1	5	33.5	43.6

Note: The data for AO, PR, DR. RR, HS, CO and RE are taken from the [15] Global Health Security Index (2019), pages 20–29, while the data for GM and HM are calculated in this paper.

## Appendix B

**Table A2:** GHS Index Ordinal Rankings for 195 countries.

Rank	AO	GM	HM	PR	DR	RR	HS	CO	RE
United States	1	1	1	1	1	1	1	1	19
United Kingdom	2	5	5	10	6	6	11	2	26
Netherlands	3	6	8	4	7	8	3	32	12
Australia	4	2	2	8	2	2	6	3	18
Canada	5	3	3	7	4	4	4	5	10
Thailand	6	8	7	3	15	15	2	12	93
Sweden	7	4	6	2	7	9	20	11	6
Denmark	8	7	10	5	7	7	5	28	17
South Korea	9	9	12	19	5	5	13	23	27
Finland	10	12	14	9	45	45	9	4	14
France	11	10	15	6	21	21	8	44	9
Slovenia	12	17	18	12	27	27	18	8	29
Switzerland	13	15	13	34	48	48	7	18	3
Germany	14	11	16	13	10	10	22	29	11
Spain	15	16	19	32	11	11	12	32	24
Norway	16	14	11	11	49	49	14	22	2
Latvia	17	13	9	25	2	3	23	79	48
Malaysia	18	25	31	35	28	29	15	45	33
Belgium	19	19	23	15	42	42	10	38	19
Portugal	20	29	33	33	61	61	17	26	22
Japan	21	23	29	40	35	34	25	13	34
Brazil	22	27	24	16	12	12	33	135	94
Ireland	23	21	25	14	18	18	41	66	21
Singapore	24	31	35	23	40	40	38	101	15
Argentina	25	24	27	66	23	23	18	14	70
Austria	26	18	17	18	28	28	25	66	5
Chile	27	33	39	23	30	30	43	78	38
Mexico	28	26	22	49	32	33	24	6	89
Estonia	29	22	28	44	19	19	66	15	30
Indonesia	30	30	26	38	37	37	42	7	106
Italy	31	28	30	45	16	16	54	29	55
Poland	32	34	40	37	44	44	21	41	45
Lithuania	33	20	20	59	13	13	63	8	46
South Africa	34	37	32	51	13	14	65	107	64
Hungary	35	39	48	22	55	53	56	41	42
New Zealand	35	42	44	27	107	107	32	39	23
Greece	37	35	34	28	17	17	50	92	80
Croatia	38	32	37	26	31	31	27	92	42
Albania	39	46	51	57	25	25	59	65	100
Turkey	40	40	42	20	74	74	30	23	92
Serbia	41	52	50	43	69	68	16	86	74
Czech Republic	42	41	49	36	60	60	52	41	28
Georgia	42	36	38	31	22	22	45	53	113
Armenia	44	54	58	21	46	46	81	83	123
Ecuador	45	51	55	29	32	32	60	126	88
Mongolia	46	43	43	73	20	20	69	72	69
Kyrgyz Republic	47	53	54	109	39	39	70	20	96
Saudi Arabia	47	44	47	89	24	24	35	81	71
Peru	49	57	60	60	102	102	33	26	84
Vietnam	50	49	53	39	51	51	74	21	107
China	51	59	65	50	64	64	30	141	58
Slovakia	52	50	57	30	70	71	48	66	36
Philippines	53	61	68	71	41	41	47	84	124
Israel	54	55	64	54	58	57	37	138	41
Kenya	55	48	45	48	36	36	103	16	155
United Arab Emirates	56	58	59	40	126	126	98	25	31
India	57	69	79	87	67	66	36	100	103
Iceland	58	56	46	84	104	104	28	128	13
Kuwait	59	70	82	68	66	66	57	132	66
Romania	60	62	70	42	85	86	55	75	53
Bulgaria	61	47	56	73	57	57	39	31	50
Costa Rica	62	60	67	53	54	53	86	129	34
Russia	63	83	89	62	116	116	50	72	113
Uganda	63	67	63	63	62	62	152	19	173
Colombia	65	71	80	75	91	92	64	35	116
El Salvador	65	64	62	150	26	26	85	82	129
Luxembourg	67	45	21	102	91	92	48	66	4
Montenegro	68	63	74	79	56	56	71	63	77
Morocco	68	80	86	88	53	53	71	170	97
Panama	68	79	87	69	78	79	61	161	60
Liechtenstein	71	38	4	61	149	149	67	48	1
Myanmar	72	72	73	106	47	47	111	40	164
Laos	73	81	76	165	34	34	112	113	133
Lebanon	73	75	83	116	43	43	92	88	134
Nicaragua	73	74	78	65	99	99	29	76	154
Oman	73	73	84	84	97	97	82	53	53
Cyprus	77	65	71	47	76	76	99	92	40
Moldova	78	68	75	46	83	84	58	50	132
Bosnia and Herzegovina	79	89	91	78	91	91	45	152	119
Jordan	80	87	101	97	83	83	79	96	99
Uruguay	81	78	72	54	119	119	89	146	25
Qatar	82	86	88	93	124	124	44	170	44
Kazakhstan	83	66	61	17	133	134	77	66	72
Ethiopia	84	76	66	77	118	118	73	17	175
Bhutan	85	93	105	83	85	85	78	145	90
Madagascar	86	108	111	107	90	90	113	55	180
Egypt	87	102	116	79	96	96	128	104	86
Bahrain	88	88	93	81	72	71	80	189	82
Cambodia	89	77	77	110	50	50	146	36	162
North Macedonia	90	90	103	76	91	94	82	119	84
Dominican Republic	91	120	127	105	105	105	125	126	73
Sierra Leone	92	94	100	128	72	73	84	66	179
Zimbabwe	92	84	81	101	38	38	132	113	158
Ukraine	94	97	104	72	109	110	97	57	146
Senegal	95	114	114	126	114	114	116	47	128
Nigeria	96	96	96	123	78	78	107	50	174
Iran	97	100	98	52	103	103	62	186	124
Malta	98	85	85	86	123	123	94	92	32
Trinidad and Tobago	99	109	106	112	170	170	93	57	58
Suriname	100	135	139	144	107	107	123	119	111
Tanzania	101	107	109	91	89	89	175	55	137
Bolivia	102	116	118	54	122	121	131	97	118
Paraguay	103	112	117	70	115	115	75	161	97
Namibia	104	106	113	96	70	70	160	122	102
Côte d'Ivoire	105	101	108	116	80	80	119	61	149
Ghana	105	122	130	95	98	98	96	148	116
Pakistan	105	125	131	136	91	95	107	86	160
Belarus	108	131	119	162	132	131	40	193	109
St. Lucia	108	126	122	147	129	128	189	60	63
Cuba	110	98	94	66	177	176	52	84	82
Liberia	111	92	52	176	131	131	107	10	170
Nepal	111	130	129	58	150	149	76	167	137
Bangladesh	113	91	92	116	59	57	132	74	142
Mauritius	114	111	110	116	87	87	130	184	51
Cameroon	115	105	95	111	113	113	101	37	175
Uzbekistan	116	104	90	64	156	156	127	34	130
Azerbaijan	117	115	120	104	75	75	118	160	104
Gambia	117	134	135	152	106	105	95	122	131
Rwanda	117	129	134	90	111	112	89	148	144
Sri Lanka	120	121	126	135	82	82	122	137	91
Maldives	121	139	140	154	138	138	117	117	79
Tunisia	122	138	137	99	136	135	91	177	100
St. Vincent and The Grenadine	123	118	107	160	154	154	114	46	65
Micronesia	124	163	166	157	171	170	115	157	108
Guatemala	125	123	123	156	63	63	155	132	126
Guinea	125	110	97	120	52	51	176	99	182
Monaco	125	82	41	180	146	147	68	161	8
Brunei	128	127	121	129	127	127	88	195	49
Togo	129	128	128	139	68	68	161	107	168
Afghanistan	130	103	102	140	77	77	102	52	191
Tajikistan	130	151	156	121	144	144	105	131	164
Niger	132	119	125	94	81	81	99	117	186
Barbados	133	124	112	92	157	158	173	111	39
seychelles	133	113	99	187	120	120	107	102	37
Belize	135	137	136	108	128	128	163	88	109
Turkmenistan	135	136	138	102	101	100	134	146	135
Guyana	137	143	149	113	155	155	144	88	121
Haiti	138	117	115	100	65	65	158	98	185
Botswana	139	133	133	152	133	133	138	107	62
San Marino	139	99	69	149	117	117	124	194	16
Eswatini (Swaziland)	139	141	145	82	138	140	188	103	127
Bahamas	142	142	141	130	152	151	177	111	67
Andorra	143	95	36	113	171	170	168	173	7
Lesotho	144	150	155	134	160	160	104	113	140
Burkina Faso	145	159	164	168	121	121	191	119	151
Cabo Verde	146	140	132	113	178	178	125	164	47
Antigua and Barbuda	147	132	124	170	157	157	181	57	56
Jamaica	147	145	146	159	142	142	161	129	68
Mali	147	144	144	142	138	139	140	64	181
Benin	150	154	150	172	143	143	191	61	147
Chad	150	156	157	145	109	109	186	110	189
Zambia	152	155	159	132	151	151	106	148	141
Mozambique	153	148	154	122	130	130	120	125	163
Malawi	154	146	151	125	146	146	129	80	168
Papua New Guinea	155	168	170	185	125	125	152	139	160
Honduras	156	161	168	155	135	135	146	136	156
Grenada	157	147	142	190	159	159	159	104	61
Mauritania	157	149	153	186	100	100	120	157	156
Central African Republic	159	176	178	168	162	161	142	122	192
Comoros	160	158	158	163	148	147	166	77	171
Congo (Democratic Republic)	161	165	167	137	141	141	150	113	194
Samoa	162	157	147	158	173	173	168	179	52
St. Kitts and Nevis	163	152	143	189	167	166	185	104	57
Sudan	163	172	172	97	185	186	135	153	178
Vanuatu	165	164	162	132	167	169	186	148	87
Timor-Leste	166	174	175	167	137	137	163	164	153
Iraq	167	153	152	150	88	88	150	183	184
Fiji	168	167	160	131	165	165	179	190	75
Libya	168	160	165	145	111	111	170	177	159
Angola	170	171	173	137	161	161	157	139	152
Tonga	171	169	163	161	167	168	179	164	76
Dominica	172	162	161	179	176	176	173	88	105
Algeria	173	170	171	124	174	174	139	184	113
Congo (Brazzaville)	173	166	148	171	185	185	189	49	167
Djibouti	175	181	184	173	164	164	167	157	149
Venezuela	176	175	177	140	182	184	141	132	164
Burundi	177	184	185	127	175	175	172	153	187
Eritrea	178	177	180	142	163	163	163	144	177
Palau	179	178	176	192	180	181	154	175	94
South Sudan	180	183	183	148	166	166	137	172	193
Tuvalu	181	173	169	178	182	183	146	187	78
Nauru	182	187	186	188	189	192	146	175	120
Solomon Islands	183	182	182	191	182	181	143	143	142
Niue	184	179	174	181	189	193	170	181	81
Cook Islands	185	180	181	182	180	180	135	181	121
Gabon	186	188	188	183	188	188	156	156	147
Guinea-Bissau	186	186	187	177	145	145	194	153	188
Syria	188	185	179	166	194	195	87	192	183
Kiribati	189	192	192	184	189	190	182	174	136
Yemen	190	190	190	175	179	179	178	141	190
Marshall Islands	191	189	189	195	189	191	183	179	112
São Tomé and Príncipe	192	194	194	192	194	194	183	167	139
North Korea	193	191	191	164	185	186	145	191	172
Somalia	194	193	193	174	153	153	195	188	195
Equatorial Guinea	195	195	195	195	189	189	193	167	144

Note: The data are derived in this paper.
